# Identifying novel biomarkers for biliary tract cancer based on volatile organic compounds analysis and machine learning

**DOI:** 10.3389/fonc.2025.1572460

**Published:** 2025-04-24

**Authors:** Jingrong Qian, Qi Liu, Jue Wang, Xuewei Zhuang, Jun Fang

**Affiliations:** ^1^ Department of Clinical Laboratory, Shandong Provincial Third Hospital, Shandong University, Jinan, Shandong, China; ^2^ Department of Medical Engineering, Shandong Province Third Hospital, Shandong University, Jinan, Shandong, China

**Keywords:** volatile organic compounds, machine learning, biliary tract cancer, novel biomarkers, SVM, LDA, KNN, LASSO

## Abstract

**Background:**

The current diagnostic methods for biliary tract cancer (BTC) have limitations in sensitivity and specificity. This study aims to explore the use of volatile organic compounds (VOCs) in serum to distinguish BTC and benign biliary diseases (BBD).

**Method:**

We collected 158 serum samples from BTC and BBD patients, and used gas chromatography ion mobility spectrometry (GC-IMS) for VOCs detection. Six machine learning methods (RF, SVM, LDA, KNN, LASSO, and XGBoost) were used to construct and evaluate diagnostic prediction models.

**Result:**

We detected a total of 40 VOCs in patients, of which 14 VOCs were statistically significant (*p* < 0.05), including 11 up-regulated and 3 down-regulated VOCs. In BTC and BBD patients, the diagnostic model was constructed based on six machine learning method. Among them, RF had the highest diagnostic performance (AUC = 0.935, *p* < 0.001), with a sensitivity of 76.2% and a specificity of 96.3%. Based on the importance score, we selected the top 4 VOCs, and constructed an optimized diagnostic model through five fold cross validation. The model’s AUC was 0.982, sensitivity was 87.9%, and specificity was 96.7%, which improved the diagnostic sensitivity and reduced FNR. In addition, in patients with cholangiocarcinoma and BBD, we further screened for 4-VOCs and constructed diagnostic model, with an AUC of 0.977, accuracy of 92.4%, specificity of 98.9%, sensitivity of 77.5%.

**Conclusion:**

The diagnostic model based on 4-VOCs may be a feasible method for distinguishing the diagnosis of BTC and BBD patients.

## 1 Introduction

Biliary tract cancer (BTC) was an invasive malignant tumor of the hepatobiliary pancreatic system, including cholangiocarcinoma, gallbladder cancer, and Vater ampulla cancer (1). At present, in developing countries, the incidence rate was increasing year by year (2). At the beginning of the disease, patients usually have no specific symptoms, leading to late diagnosis. The main treatment scheme of BTC relies on surgical resection plus adjuvant chemotherapy, but the recurrence rate was still high (3). The 5-year survival rate of patients was between 5% and 15% (4, 5). Therefore, it was crucial to identify novel and effective diagnostic biomarkers for BTC.

Human volatile organic compounds (VOCs) was an ultimate metabolite, which reflect the metabolic changes caused by external and internal factors (such as inflammation, necrosis and disease, including cancer), and can be detected in exhaled breath, blood, urine and other body fluid samples (6). At present, more and more attention has been paid to the development of diagnostic markers based on its detection. VOCs has been confirmed to have content changes in diabetes, infectious diseases, lung cancer, breast cancer, pancreatic cancer and other tumors, and has been explored as a biomarker (7–9). It was worth noting that the analysis of VOCs in urine and bile samples has shown good sensitivity and specificity in exploring biomarkers for malignant biliary stenosis (10, 11).

Serum was a biological sample containing a large amount of cellular metabolism and was easy to collect and store. However, there were currently no studies reporting the analysis of VOCs in serum to discover novel biomarkers for BTC. This study aims to explore the change in VOCs in the serum of patients with BTC and benign biliary diseases (BBD), and to construct a novel diagnostic model for BTC patients through machine learning.

## 2 Method

### 2.1 Research population

**Figure 1:**
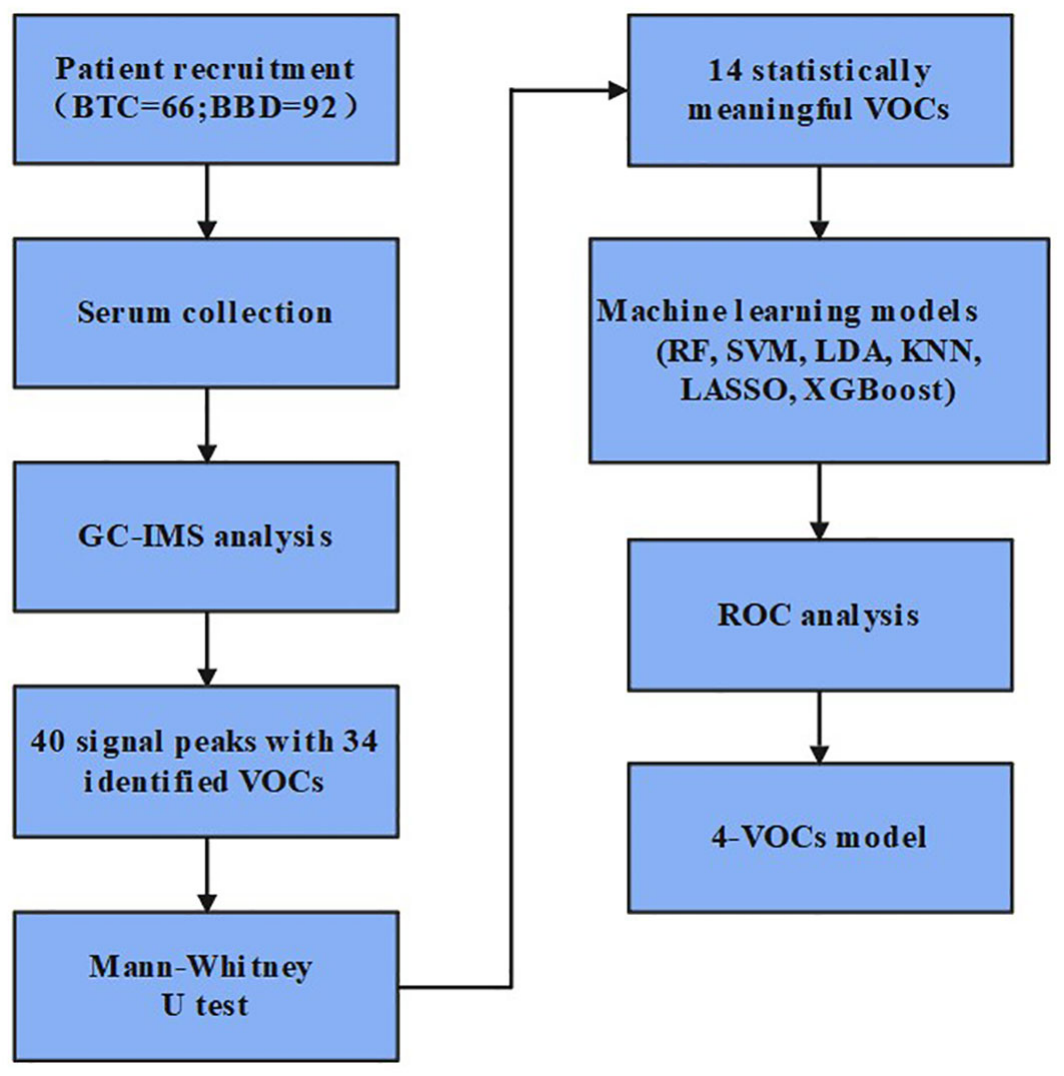
Design flowchart for this study.

As shown in **Figure 1**, this study included 66 BTC patients and 92 BBD patients who visited the Third Hospital of Shandong Province from January to August 2024. The inclusion criteria for BTC patients were: (1) patients who have not been diagnosed with other tumors or received tumor treatment before seeking medical attention; (2) the final result of the patient’s pathological diagnosis shall be determined by at least two pathologists; (3) complete clinical information; The inclusion criteria for BBD patients were: (1) diagnosis of benign biliary obstruction based on clinical symptoms, imaging examination, ERCP, or pathological examination; (2) first diagnosis; (3) complete clinical information. This study has been approved by the Ethics Committee of Shandong Third Hospital of Shandong University (No.KYLL-2023084).

### 2.2 Samples

Extract 5mL of fasting peripheral venous blood from the patient, centrifuge at 3000 r/min for 10 minutes, separate the supernatant, divide it, and store it at -80°C. We used the fully automated chemiluminescence analyzer (Beckman, AU5800) to measure alanine aminotransferase (ALT, U/L), aspartate aminotransferase (AST, U/L), mitochondrial aspartate aminotransferase (mAST, U/L), γ-glutamyl transferase (γ-GGT, U/L), alkaline phosphatase (ALP, U/L), gluathione reductase (GR, U/L), albumin (ALB, g/L), total bilirubin (TBIL, umol/L), direct bilirubin (DBIL, umol/L), indirect bilirubin (IBIL, umol/L), and total bile acod (TBA, umol/L).

### 2.3 VOCs detection

We used gas chromatography ion mobility spectrometry (GC-IMS, Dortmund Gas, Germany) instrument to detect VOCs in serum samples (12). The sample was heated in an incubator and placed in a sample tray. The sample was extracted by an injector and separated once in gas chromatography, and then separated twice in ion migration spectra. Due to the different mass, charge, collision interface, and spatial configuration of the sample, their migration rates in the electric field were different, and the time they arrive at the detector was also different. The detector collects the ion signal to form a gas phase ion migration spectrum, and analyzes it to obtain the substance content.

The specific testing information was as follows: 1) Take 200 microliters of serum and place it in a headspace vial, incubate at 55 °C for 5 minutes. 2) The temperature of HS syringe was 85°C, the injector temperature was 80°C, the injection was splitless. Extract 1 milliliter of headspace gas for analysis; 3) The program parameter settings are as follows: Nitrogen is used as the carrier gas. The IMS drif gas flow rate is maintained at 75 mL/min, and the carrier gas gradient is as follows: 0 min: 2 mL/min; 1 minute: 2 milliliters per minute; 8 minutes: 100 milliliters per minute; 10 minutes: 150 milliliters per minute; 15 minutes: 150 milliliters per minute. T1 diversion pipe temperature: 45°C; T2 gas chromatography column temperature: 60°C; Inlet temperature of T3 chromatographic column converter: 80°C; T4 connection line 1: 80°C, T5 connection line 2: 45°C. Ionic mode: Positive ion mode. The chromatographic column model was MXT-5 (Restek Company), which was a stainless steel capillary column coated with fused silica. Its stationary phase was cross-linked bonded 5% diphenyl, 95% dimethyl polysiloxane, with an inner diameter of 0.53 mm, a film thickness of 1 micron, and a length of 15 meters.

### 2.4 VOCs identification

The detected VOCs were identified based on the retention index (RI) of gas chromatography (GC) and the relative migration time (Drift time. Dt) of ion mobility spectrometry (IMS). The RI data was obtained from the NIST database (NIST 2020RI), and the Dt data was obtained from the self built IMS database (Hanon 2024 IMS). The peak position of volatile organic compounds was confirmed by comparing it with the peak position of the standard substance (n-ketones of C4-C9), and the RI and Dt of the test substance must be consistent with the data of the standard substance. The requirement for the compound signal on the spectrum was that the three-dimensional signal on the spectrum should have a regular peak shape, and its peak intensity should be at least three times greater than the baseline noise. In addition, by normalizing RIP, the deviation in absolute migration time of ions can be eliminated. All names of volatile organic compounds were taken from the NIST spectral library. In chemistry, these naming conventions were also typical and can be accurately defined by the CAS number (Chemical Abstracts Service, abbreviated as CAS), an organization under the American Chemical Society. Use peak intensity for semi quantitative analysis of substances, the unit was volt (V). We used the supporting VOCal analysis software and Reporter, Gallery Plo plugins to analyze the data.

### 2.5 Statistical analysis

The classified data in the clinical information of patients in this study were displayed as frequency and percentage, and the continuous data were displayed as median (25% numerical value, 75% numerical value). The categorical data were compared using the χ2 square test, and the continuous data between the two groups were compared using the Mann Whitney U test. A *p* -value < 0.05 was considered statistically significant; Use IBM SPSS software (version 22.0) and GraphPad Prism (version 8.3.0) for data analysis.

We used six machine learning methods including RandomForest (RF), Support Vector Machine (SVM), Latent Dirichlet Allocation (LDA), K-Nearest Neighbors (KNN), Least Absolute Shrinkage and Selection Operator (LASSO), and Extreme Gradient Boosting (XGBoost) to construct a diagnostic model. Each method randomly divided 70% of patients into a training set and 30% into a validation set, plotted Receiver Operating Characteristic (ROC) curves, and calculated accuracy {[True Positive (TP) + True Negative (TN)]/TP + TN + False Positive (FP) + False Negative (FN)}, precision (TP/TP + FP), sensitivity (TP/TP + FN), specificity (TN/TN + FP), false positive rate (FPR = FP/FP + TN), false negative rate (FNR = FN/TP + FN). The *p* - value < 0.05 was considered statistically significant. In the random forest method, out of bag data (OOB) was used to calculate feature importance. The higher the score, the greater the role of the substance in the decision-making process of the random forest model. We used grid search to call GridSearchCV from sklearn. Each set of parameter combinations was performed five fold cross validation, and selected the best super parameter combination to achieve the effect of model optimization.

## 3 Result

### 3.1 Clinical characteristics of patients

The average age of patients with BTC included in this study was 67 years, with 50% male and 50% female. Compared with patients with BBD, patients with BTC had higher levels of AST (*p* = 0.026), mAST (*p* = 0.042), γ-GGT (*p* < 0.001), ALP (*p* < 0.001), GR (*p* < 0.001), ALB (*p* < 0.001), TBIL (*p* < 0.001), DBIL (*p* < 0.001), and TBA (*p* < 0.001). However, there was no difference in ALT and IBIL levels between BTC patients and BBD patients (**Table 1**).

**Table 1:** The baseline characteristic of the patients.

	BBD	BTC	*p - value*
Age (years)	47 (19 - 88)	67 (23 - 87)	< 0.001
Gender			0.224
Male	37 (40.2%)	33 (50%)	
Female	55 (59.8%)	33 (50%)	
ALT	36.6 (16.3 - 30.8)	92.8 (23.9 - 172.8)	0.444
AST	26.4 (16.5 - 137.5)	76.4 (26.1 - 159.1)	0.026
mAST	5.8 (3.6 - 25.8)	13.0 (5.7 - 30.4)	0.042
Γ-GGT	104.0 (25.0 - 343.0)	269.0 (73.5 - 759.0)	< 0.001
ALP	97.0 (70 - 192.5)	385.0 (119.0 - 637.5)	< 0.001
GR	74.0 (59.2 - 107.6)	99.5 (74.9 - 124.9)	< 0.001
ALB	44.2 (38.9 - 46.7)	37.9 (33.5 - 42.6)	< 0.001
TBIL	14.5 (8.9 - 43.7)	56.5 (11.9 - 198.2)	< 0.001
DBIL	6.5 (4.2 - 25.2)	53.4 (6.3 - 180.7)	< 0.001
IBIL	6.6 (4.3 - 10.7)	7.6 (3.9 - 19.2)	0.329
TBA	3.8 (1.7 - 31.8)	26.7 (4.4 - 153.3)	< 0.001

BBD, benign biliary diseases; BTC, biliary tract cancer; ALT, alanine aminotransferase; AST,aspartate aminotransferase; mAST, mitochondrial aspartate aminotransferase; γ-GGT, γ-glutamyl transferase; ALP, alkaline phosphatase; GR, gluathione reductase; ALB, albumin; TBIL, total bilirubin; DBIL, direct bilirubin; IBIL, indirect bilirubin; TBA, total bile acod.

### 3.2 Analysis of VOCs

**Figure 2:**
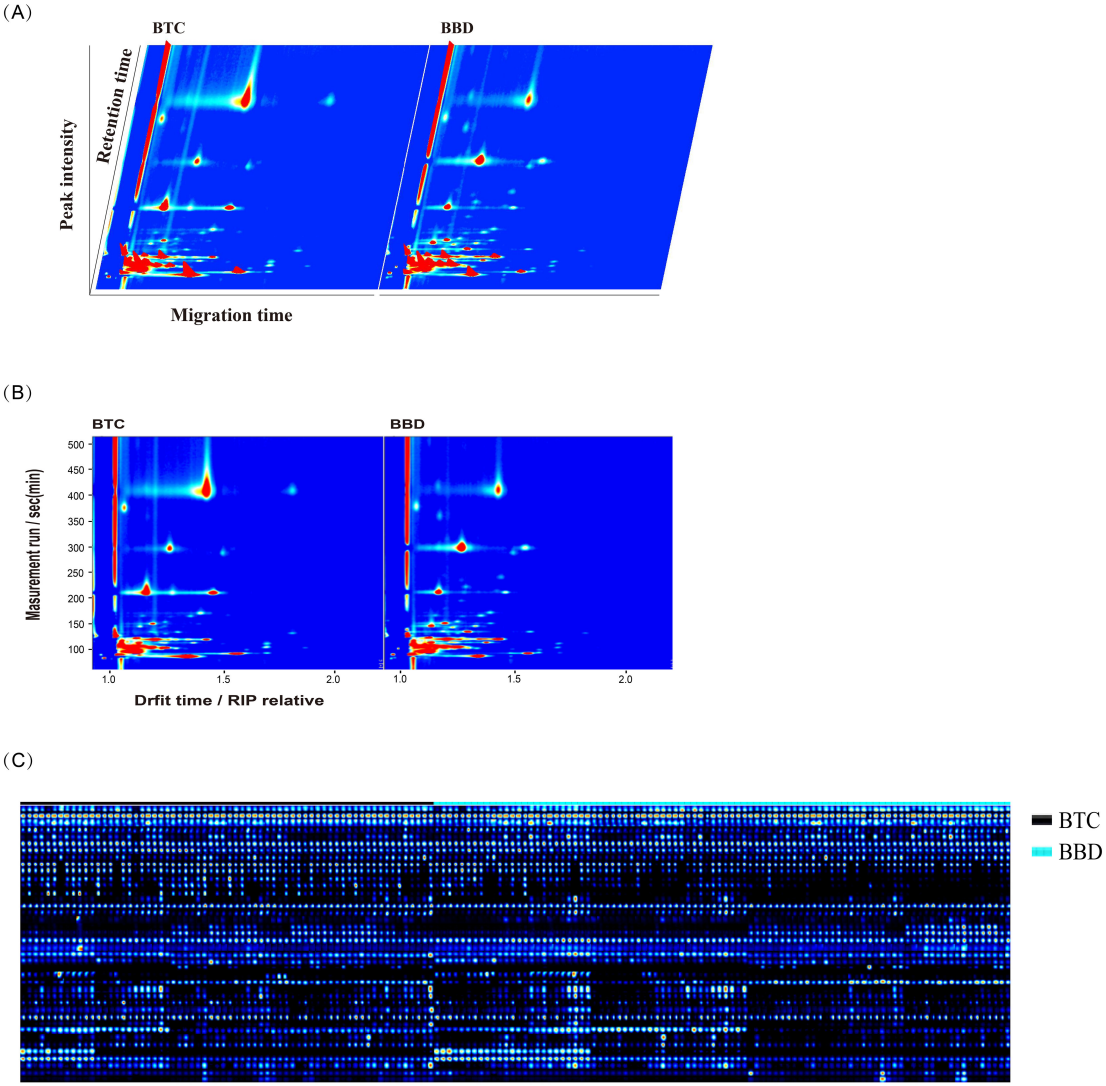
Characteristics of volatile organic compounds (VOCs) detected in BBD and BTC patients. **(A)** The three-dimensional spectrum consisting of gas retention time, ion migration time, and signal peak intensity; **(B)** The two-dimensional spectrum shows the migration time and retention index of different VOCs; **(C)** Peak signals of VOCs detected in BBD and BTC patients.

We used GC-IMS technology to analyze VOCs in the serum of BTC patients and BBD patients. **Figure 2A** shows a three-dimensional spectrum consisting of gas retention time, ion migration time, and signal peak intensity. The VOC signal peaks of each sample were also characterized. **Figure 2B** shows a two-dimensional spectrum, which was a vertical view of a three-dimensional spectrum, with peak intensity represented by colors, displaying the difference in VOC signal peaks in BTC patients and BBD patients. Based on the above analysis, a total of 40 VOCs were detected in the serum of BTC and BBD patients (**Figure 2C**), including 34 VOCs and 6 unknown VOCs. **Supplementary Table 1** provides detailed information on these compounds, including name, CAS number, Formula molecular formula, molecular weight (MW), retention index (RI), retention time (Rt), migration time (Dt) and detection frequency.

### 3.3 Differential analysis of VOCs between BTC and BBD patients

**Figure 3:**
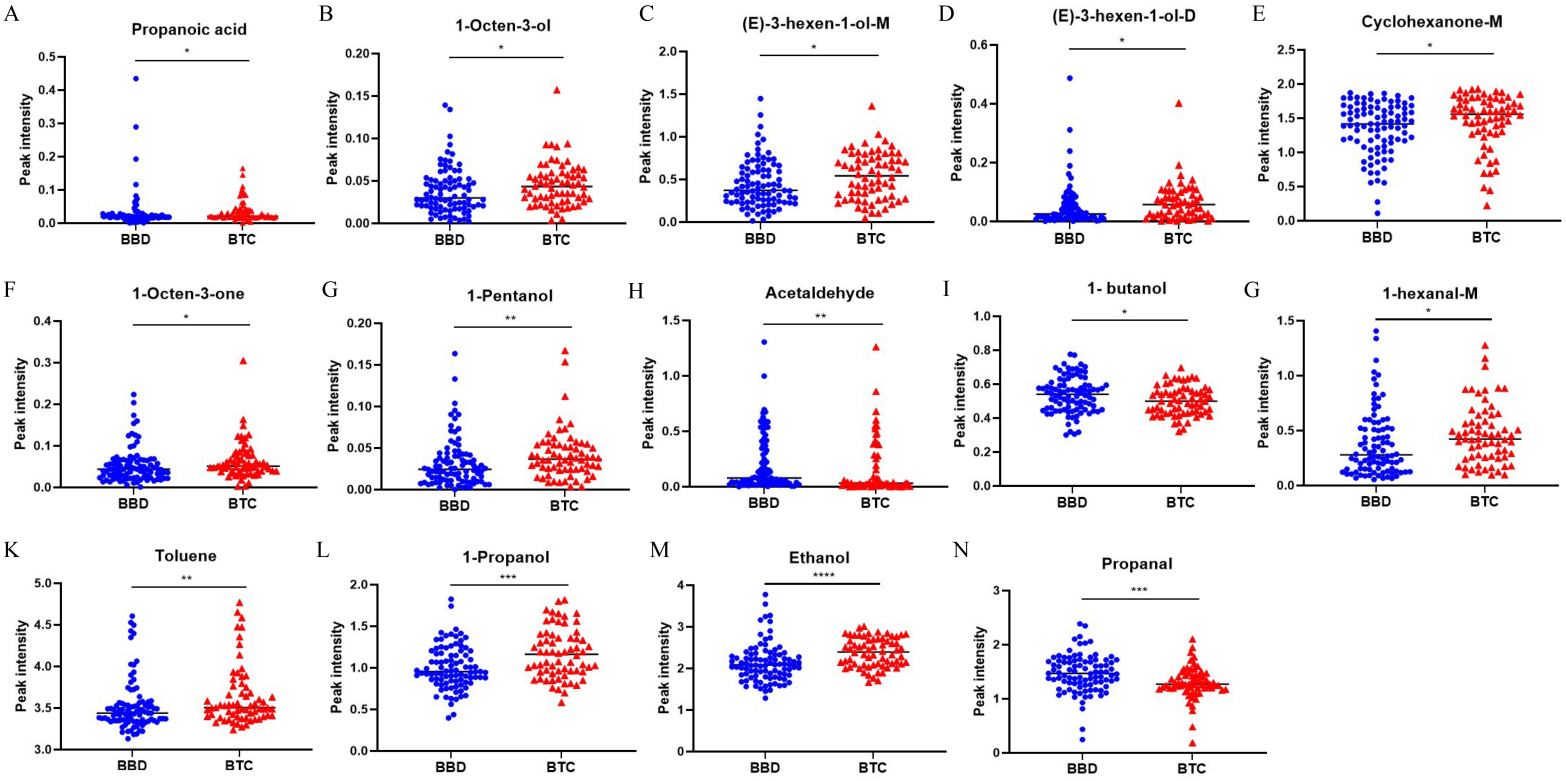
Significant difference analysis of VOCs peak intensity between BTC patients and BBD patients. *p < 0.05, **p < 0.01, ***p < 0.001 ****p < 0.0001 (Mann–Whitney U-test).

Compared with BBD patients, we found that there were significant differences in the peak intensity of 14 VOCs in BTC patients. (*p* < 0.05, **Table 2**, **Figure 3**), among which 11 VOCs were up-regulated, including Ethanol (2.396 vs. 2.085, *p* < 0.001), 1-Propanol (1.162 vs. 0.954, *p* = 0.001), 1-Pentanol (0.037 vs. 0.025, *p* = 0.004), Toluene (3.504 vs. 3.439, *p* = 0.009), and 1-Octen-3-one (0.051 vs. 0.044, *p* = 0.011). Three VOCs were down regulated, including Propanol (1.270 vs. 1.469, *p* < 0.001). Acetaldehyde (0.032 vs. 0.078, *p* = 0.004) and 1-butanol (0.500 vs. 0.540, *p* = 0.026). Subsequently, we included these 14 VOCs for further model construction. In addition, we further demonstrated the fingerprint spectra of these 14 characteristic peaks in patients (**Supplementary Figure 1**).

**Table 2:** Comparisons of peak intensity of VOCs in BBD patients and BTC patients.

VOCs	BBD	BTC	*p - value*
Propanoic acid	0.018 (0.016 - 0.023)	0.021 (0.017 - 0.040)	0.022
1-Octen-3-ol	0.030 (0.019 - 0.050)	0.043 (0.027 - 0.058)	0.018
(E)-3-hexen-1-ol-M	0.372 (0.235 - 0.611)	0.543 (0.272 - 0.747)	0.017
(E)-3-hexen-1-ol-D	0.025 (0.013 - 0.064)	0.057 (0.017 - 0.101)	0.031
Cyclohexanone-M	1.415 (1.147 - 1.659)	1.556 (1.301 - 1.764)	0.026
1-Octen-3-one	0.044 (0.023 - 0.066)	0.051 (0.039 - 0.078)	0.011
1-Pentanol	0.025 (0.011 - 0.042)	0.037 (0.024 - 0.054)	0.004
Acetaldehyde	0.078 (0.034 - 0.355)	0.032 (0.011 - 0.274)	0.004
1- butanol	0.540 (0.450 - 0.595)	0.500 (0.431 - 0.560)	0.026
1-hexanal-M	0.279 (0.147 - 0.520)	0.424 (0.260 - 0.599)	0.012
Toluene	3.439 (3.349 - 3.561)	3.504 (3.399 - 3.755)	0.009
1-Propanol	0.954 (0.841 - 1.174)	1.162 (0.952 - 1.369)	0.001
Ethanol	2.085 (1.827 - 2.329)	2.396 (2.103 - 2.703)	<0.001
Propanal	1.469 (1.266 - 1.698)	1.270 (1.191 - 1.440)	<0.001

VOCs, volatile organic compounds; BBD, benign biliary diseases; BTC, biliary tract cancer.

### 3.4 Construction of diagnostic models using machine learning

**Figure 4:**
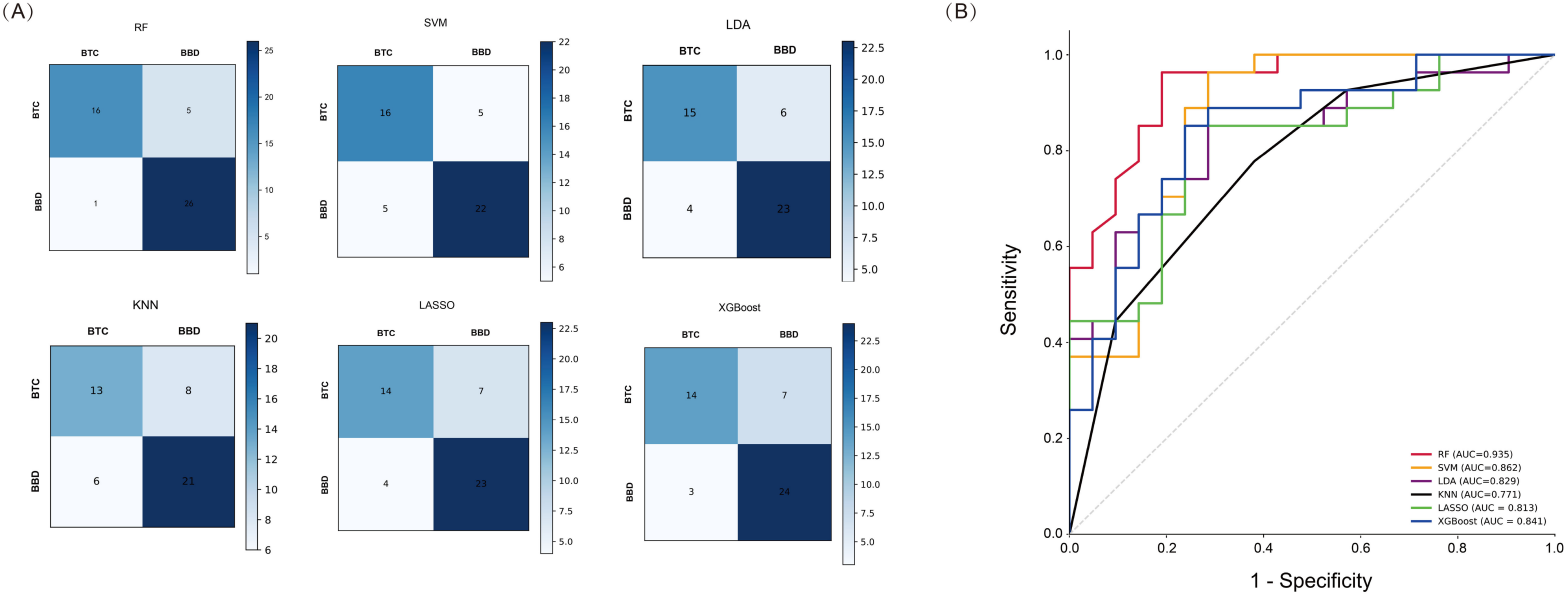
Performance Analysis of Machine Learning in the validation set. **(A)** Confusion matrix of six machine learning methods; **(B)** ROC curves of six machine learning methods.

We used six machine learning methods (RF, SVM, LDA, KNN, LASSO, and XGBoost) to construct a diagnostic model for BTC patients. Each method randomly divided 70% of patients into a training set and 30% into a validation set. We used the training set to construct a diagnostic prediction model and evaluated the diagnostic performance of the model using the validation set (**Figure 4A**). We found that the machine learning models constructed by these six machine learning methods all had good diagnostic efficiency (*p* < 0.05, **Table 3**, **Figure 4B**). Among them, RF had the highest diagnostic performance (AUC = 0.935, *p* < 0.001), sensitivity of 76.2%, specificity of 96.3%, precision rate of 94.1%, FPR of 3.7%, and the FNR of 23.8%.

**Table 3:** The AUC, sensitivity and specificity of machine learning model.

Model	AUC	Sensitivity (%)	Specificity (%)	Accuracy (%)	Precision (%)	FPR (%)	FNR (%)	*p - value*
RF	0.935	76.2	96.3	87.5	94.1	3.7	23.8	< 0.001
SVM	0.862	76.2	85.2	81.3	80.0	14.8	23.8	< 0.001
LDA	0.829	71.4	85.2	79.2	78.9	14.8	28.6	< 0.001
KNN	0.771	61.9	77.8	70.8	68.4	22.2	38.1	0.001
LASSO	0.815	85.2	66.7	77.1	76.7	33.3	14.8	< 0.001
XGBoost	0.841	66.7	88.9	79.2	82.4	11.1	33.3	< 0.001

AUC, the area under the curve;FPR, false positive rate; FNR, false negative rate; RF, RandomForest; SVM, Support Vector Machine; LDA, Latent Dirichlet Allocation, KNN, K-Nearest Neighbors, LASSO, Least Absolute Shrinkage and Selection Operator, XGBoost, Extreme Gradient Boosting.

**Figure 5:**
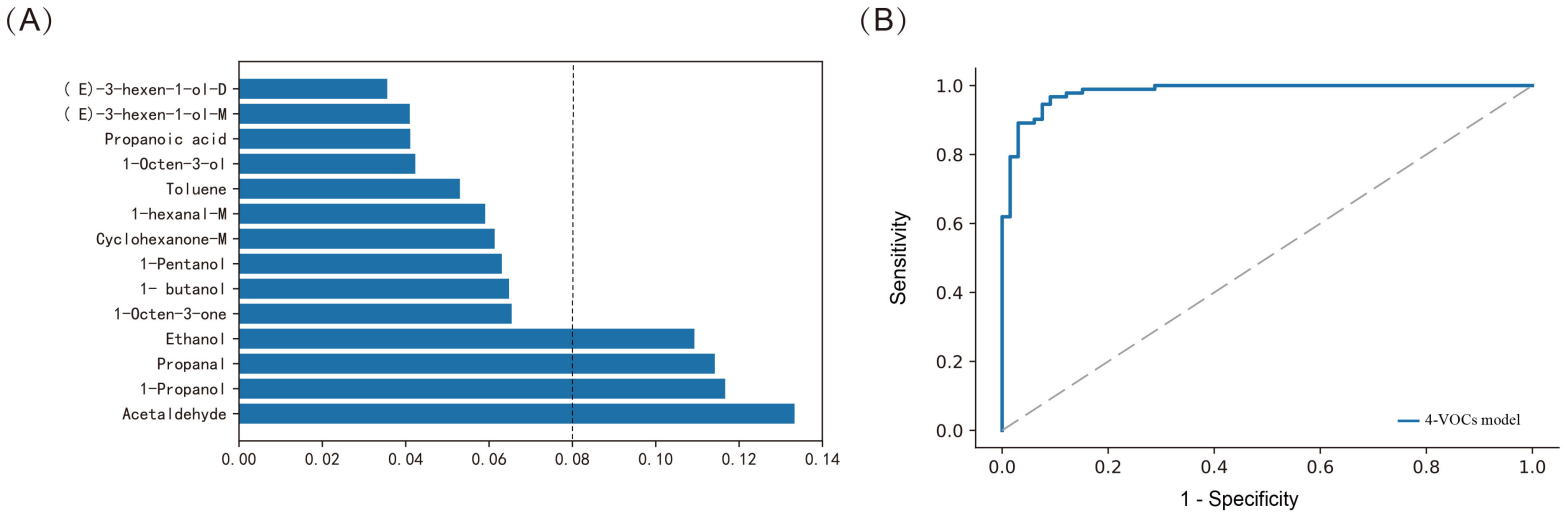
Constructing the model using Random Forest method for distinguishing between BTC and BBD patients. **(A)** Assessing the importance of VOCs; **(B)** ROC curves of 4-VOCs model.

According to the RF model, we further ranked the importance of VOCs in the model. Based on the importance score, we selected the top 4 VOCs (**Figure 5A**), which were Acetaldehyde, 1-Propanol, Propanal and Ethanol. We optimized the model by adjusting parameters and conducting five fold cross validation on each parameter combination using online search. We constructed a novel diagnostic model with an AUC of 0.982, sensitivity of 87.9%, specificity of 96.7%, and FNR of 12.1% (*p* < 0.001, **Figure 5B**). This model improved the sensitivity of diagnosis while reducing FNR.

**Figure 6:**
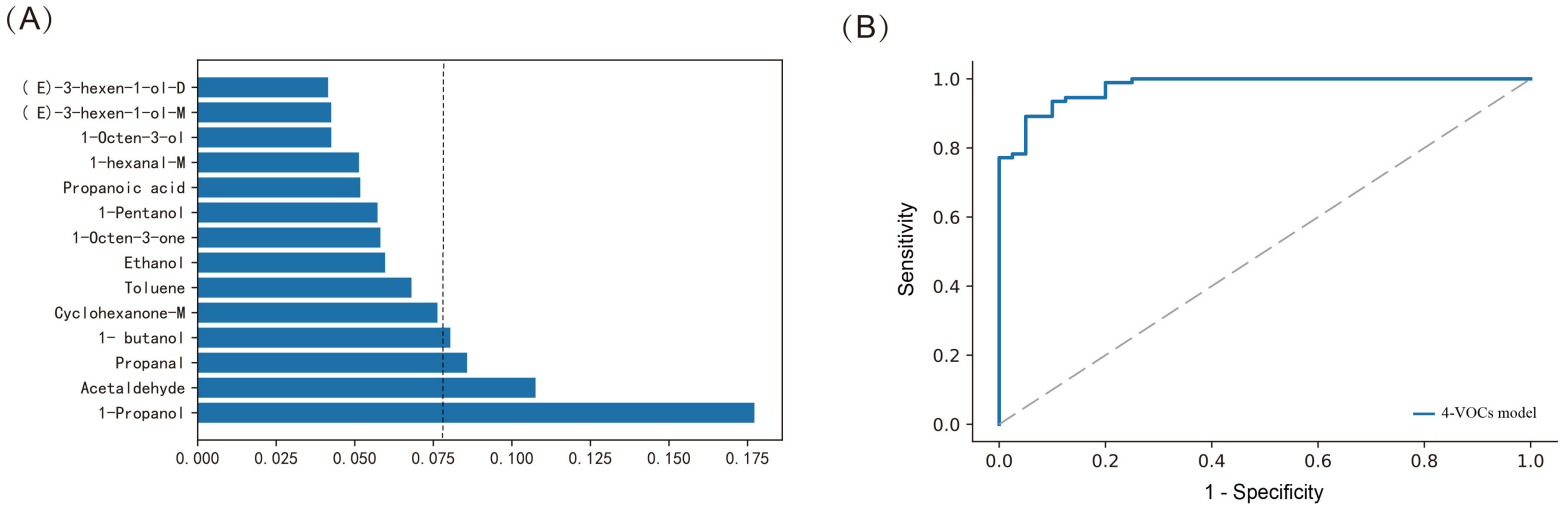
Constructing the model using Random Forest method for distinguishing between cholangiocarcinoma and benign biliary diseases patients. **(A)** Assessing the importance of VOCs; **(B)** ROC curves of 4-VOCs model.

Meanwhile, we further used the Random Forest (RF) method to construct a diagnostic model based on 14-VOCs to distinguish between cholangiocarcinoma and benign biliary diseases. The AUC of the model was 0.872, the accuracy was 82.5%, the specificity was 92.9%, and the sensitivity was 58.3% (*p* < 0.001). Further analyze the importance of VOCs in the RF model, and select the top four VOCs based on their importance ranking, namely 1-Propanol, Acetaldehyde, Propanal, and 1-butanol (**Figure 6A**). Furthermore, by adjusting the parameters and conducting five fold cross validation using online search for each parameter combination, we optimized the diagnostic model and constructed a new diagnostic model based on 4-VOCs with an AUC of 0.977, accuracy of 92.4%, specificity of 98.9%, sensitivity of 77.5%, and FPR of 1.1% (*p* < 0.001, **Figure 6B**), greatly improving the sensitivity and specificity of the model and reducing FNR and FPR.

### 3.5 Sex and age effects in model

**Figure 7:**
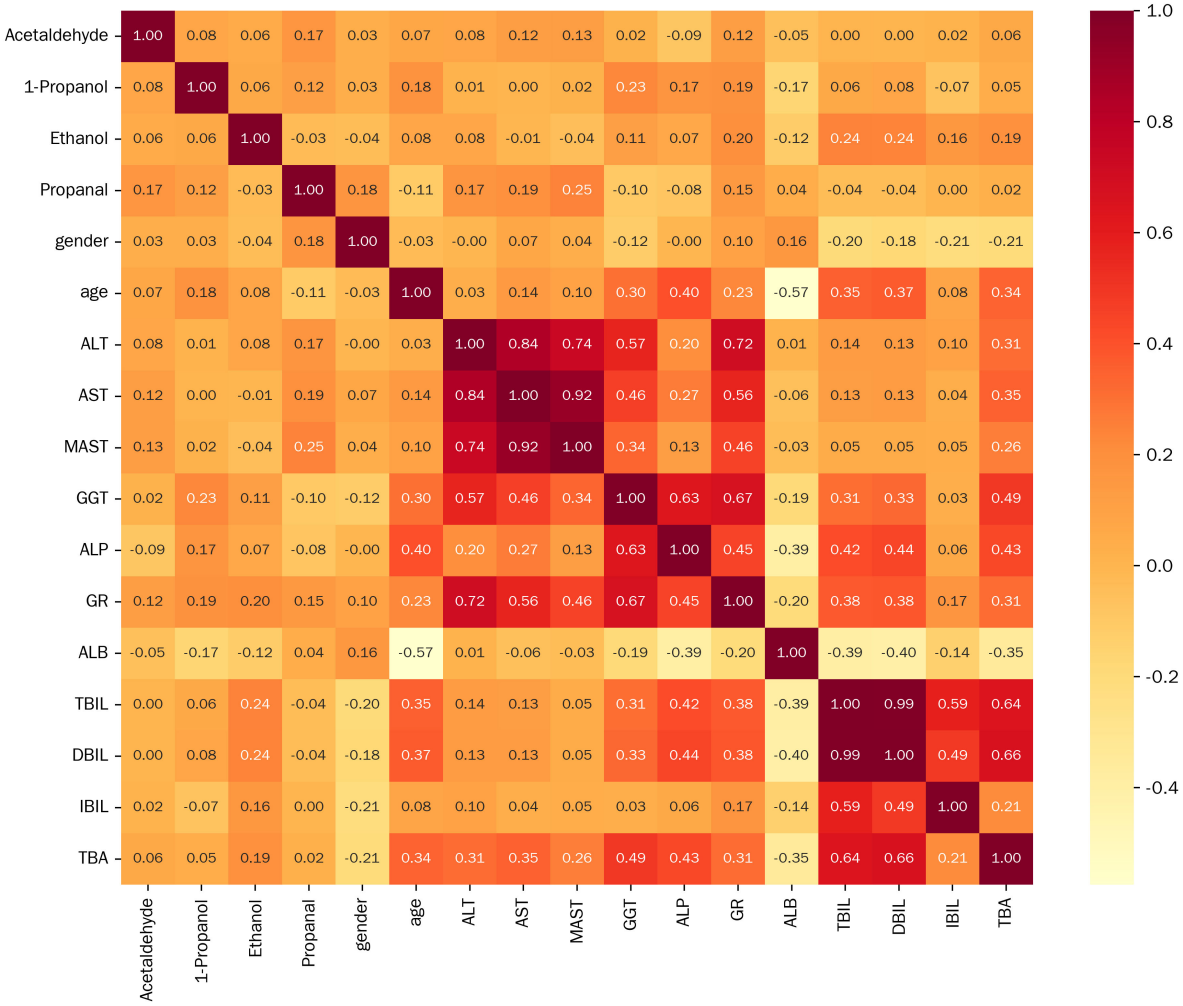
Correlation analysis heatmap between VOCs and clinical parameters.

We further conducted gender group comparison, indicator correlation, and diagnostic performance analysis of multiple models in this study. Among the included patients, we found that 88 cases were female and 70 cases were male, Proponal intensity was higher in females than males (1.431 vs. 1.311, *p* = 0.022). Subsequently, we conducted further correlation analysis (**Figure 7**), and heatmap showed the correlation values, represented by color intensity. We found that among all included patients, Propanal was related to gender (0.18, *p* = 0.027). We also found a positive correlation between 1-Propanol intensity and age (0.18, *p* = 0.023). In addition, we found that 1-Propanol was associated with patients’ GGT, ALP, GR, and ALB (*p* < 0.05), Ethanol was associated with GR, TBIL and DBIL (*p* < 0.05), Proponal was correlated with ALT and MAST (*p* < 0.05).

To further investigate the impact of gender and age on the final model, we included gender and age in the final model. In BTC and BBD patients, we found that increasing age improved the diagnostic performance of the model (**Table 4**). Compared with the 4-VOCs model, the AUC in Model 1 (4-VOCs+age) increased (0.991), while sensitivity (95.5%), specificity (97.8%), and accuracy (96.8%) were all improved, FNR and FTR were both reduced. However, the diagnostic performance of Model 2 (4-VOCs+gender) did not improve after adding gender.

**Table 4:** Diagnostic performance of different model used to distinguish between biliary tract cancer and benign biliary diseases.

Model	AUC	Accuracy (%)	Precision (%)	Sensitivity (%)	Specificity (%)	FPR (%)	FNR (%)	*p* - value
4-VOCs	0.982	0.93	0.951	0.879	0.967	0.033	0.121	< 0.001
Model 1	0.991	0.968	0.969	0.955	0.978	0.022	0.045	< 0.001
Model 2	0.979	0.918	0.949	0.848	0.967	0.033	0.152	< 0.001
Model 3	0.992	0.956	0.94	0.955	0.957	0.043	0.045	< 0.001

VOCs, volatile organic compounds; 4-VOCs include, Acetaldehyde, 1-Propanol, Propanal and Ethanol; Model 1, 4-VOCs+age; Model 2, 4-VOCs+gender; Model 3, 4-VOCs+age+gender; AUC, the area under the curve; FPR, false positive rate; FNR, false negative rate.

In addition, We also evaluated the impact of incorporating gender and age into the final model for distinguishing cholangiocarcinoma and BBD patients (**Table 5**). We found that the performance of the model changed after adding age. In Model 3 (4-VOCs+gender+age), the AUC was as high as 0.989, further improving accuracy (94.7%) and sensitivity (87.5%), and reducing FNR (12.5%), but the specificity slightly decreased (97.8%).

**Table 5:** Diagnostic performance of different model used to distinguish between cholangiocarcinoma and benign biliary diseases.

Model	AUC	Accuracy (%)	Precision (%)	Sensitivity (%)	Specificity (%)	FPR (%)	FNR (%)	*p* - value
4-VOCs	0.977	0.924	0.969	0.775	0.989	0.011	0.225	< 0.001
Model 1	0.989	0.947	0.946	0.875	0.978	0.022	0.125	< 0.001
Model 2	0.974	0.902	0.935	0.725	0.978	0.022	0.275	< 0.001
Model 3	0.989	0.947	0.946	0.875	0.978	0.022	0.125	< 0.001

VOCs, volatile organic compounds; 4-VOCs include:1-Propanol, Acetaldehyde, Propanal, and 1-butanol; Model 1, 4-VOCs+age; Model 2, 4-VOCs+gender; Model 3, 4-VOCs+age+gender; AUC, the area under the curve; FPR, false positive rate; FNR, false negative rate.

## 4 Discussion

We analyzed for the first time the VOCs in the serum of BTC and BBD patients, and applied machine learning to construct and evaluate diagnostic prediction model. Based on importance scores, we identified four VOCs. The prediction model constructed based on these four VOCs has good sensitivity and specificity, which may provide a new diagnostic basis for BTC patients.

Volatile organic compounds (VOCs) were endogenous products of cellular metabolic activity under physiological and pathological conditions, which can be detected in exhaled breath, blood, urine, and other bodily fluids. More and more studies have shown that VOCs seem to be a promising non-invasive diagnostic biomarker for cancer patients (13). The pathological mechanisms of VOC production in cancer patients include oxidative stress, cytochrome P450, carbohydrate metabolism (such as glycolysis or gluconeogenesis pathways), lipid metabolism, and loss of tumor suppressor genes, angiogenesis, or cell apoptosis, leading to significant increases or decreases in volatile organic compounds (VOCs), which may be associated with cancer diagnosis (14).

VOCs were also used as potential biomarkers for detecting gastrointestinal tumors (15). Xinru Gui et al. used GC-IMS to analyze VOCs in bile samples and found that compared with BBD patients, there were 12 differentially expressed VOCs in patients with perihilar cholangiocarcinoma (PHCCA), including class 1 alcohols, 2 ketones, 3 esters, 5 aldehydes, and 2-methoxyfuran. The diagnostic model based on 12-VOCs had good diagnostic performance with specificity of 100% and sensitivity of 93.1% (12). Udayakumar Navanethan et al. used selected ion flow tube mass spectrometry to analyze the concentrations of 22 common volatile organic compounds in bile samples, and performed logistic regression analysis to adjust for age and gender based on VOC levels of acrylonitrile, 3-methylhexane, and benzene, developing a predictive diagnostic model for cholangiocarcinoma (CCA) in patients with primary sclerosing cholangitis (PSC) (16). The above study used bile samples for analysis, while serum samples seem to be easier to obtain and store. In this study, we used GC-IMS to detect VOCs in the serum of BTC and BBD patients, and constructed and evaluated a diagnostic prediction model using machine learning with good sensitivity and specificity. According to the RF model, we identified four VOCs with high scores, including two aldehydes and two alcohols. Numerous researchers have also found ethanol and propanol in urine and bile samples of malignant biliary tumors. The increase or decrease of VOCs may be related to various metabolic pathways. Research has shown that changes in the metabolic status of CCA, including ethanol biosynthesis, pyrimidine metabolism, methanol biosynthesis, and TCA cycle, were closely related to diseases (17). In addition, the metabolic mechanism of aldehydes may include the following pathways: ADHs or cytochrome P450 CYP2E1 mediated reversible oxidation of alcohols and lipid peroxidation (18). The peroxidation of fatty acids produced under oxidative stress conditions is related to the formation of straight chain C3-C10 aldehydes (19, 20). The study reported that the occurrence and development of biliary tract tumors are closely related to lipid oxidation. Khenjanta, Chakkaphan et al. reported that cytochrome P450 enzyme (CYP39A1) and its transcription factor (RUNX2) are associated with expression and progression in cholangiocarcinoma (21). Therefore, the production of aldehydes was closely related to the occurrence and development of biliary tract tumors. We will further explore the mechanisms underlying the relationship between VOCs and the occurrence and development of biliary tract tumors.

The clinical parameters analyzed in this study reflect the liver function damage, bile stasis, and metabolic disorders of patients. The differential analysis of these clinical parameters provided a potential basis for distinguishing malignant biliary tumors from benign biliary diseases by analyzing VOCs. When conducting volatile organic compound analysis, combining these clinical parameters can provide a more comprehensive understanding of the relationship between disease status and volatile organic compounds. We found that ethanol was related to GR, which was consistent with previous research reports. Research has found that many ethanol induced pathologies are associated with oxidative stress (22). GR (glutathione reductase) was involved in the intracellular antioxidant defense system. In biliary diseases, the activity of GR may change due to an increase in oxidative stress response. Malignant tumors of the biliary tract may trigger stronger oxidative stress, leading to differences in GR levels compared to benign biliary disease groups. This explains the correlation between ethanol and GR. We will further explore the relevant mechanisms to provide new ideas and directions for the diagnosis and differential diagnosis of biliary diseases. In addition, demographic factors such as age and gender may also affect VOCs. Mar í a-Pilar Mart í nez Moral et al. used Non targeted SPME-GC/MS to explore VOCs in serum samples of pancreatic cancer. They also found that different ages and genders were related to VOCs signal intensity (23). Elina Gashimova et al. used GC-MS to detect VOCs in exhaled breath samples from lung cancer patients and healthy subjects of different ages. They analyzed and compared the peak areas of VOCs and constructed diagnostic models using various machine learning methods. They found that the diagnostic performance created using healthy subjects of different ages was roughly the same, but it was important to select parameters related to disease status rather than age (24). We found that the diagnostic performance of the model slightly increased with increasing age, and we will further expand the sample size to explore and validate the impact of population factors on VOCs.

In recent years, the application of machine learning in tumor diagnosis and treatment management has received increasing attention. In terms of modeling, machine learning can robustly analyze data and make wise judgments with minimal human involvement, thereby achieving good specificity and sensitivity in diagnostic model (25). Supervised learning trains machines using correctly classified labeled data, and then provided test data to the machines for evaluation using any supervised algorithm, resulting in accurate results (26). In this study, multiple machine learning methods were used, including RF, SVM, LDA, KNN, LASSO, and XGBoost. The machine randomly selected 70% of patients as the training set and 30% of patients as the validation set to construct and evaluate a diagnostic prediction model. It was worth noting that model validation was the process of evaluating the accuracy of a machine learning model trained on a dataset, in order to improve data quality and quantity, and ensure that the model was trustworthy before relying on its predictions. Model validation has various methods, including training/testing splitting, K-fold cross validation, leave one method cross validation, and nested cross validation (27). In this study, based on the AUC and *p* - values obtained from machine learning, we found that the model constructed using RF had the highest diagnostic efficiency. Based on the importance score, we selected the top ranked VOCs and further used network search to perform five fold cross validation on each parameter combination, thus constructing an optimized diagnostic model. Compared with previous models, this model further improved the sensitivity of diagnosis and reduced FNR. A diagnostic model based on random forest (RF) algorithm using microorganisms in tissues and blood has shown excellent performance in over 20 types of cancer (28). Xu et al. reported that ML models based on optimal algorithms improve the accuracy of cancer diagnosis by analyzing blood substances (29). We will also expand the research cohort to further evaluate our research findings.

Our research also has some limitations. 1) The research population was not included in the healthy population. The focus of this study was to identify specific biomarkers for distinguishing between patients with biliary tract tumors and benign biliary diseases, in order to better understand the pathophysiological mechanisms of disease occurrence and development, as well as to search for effective indicators for disease diagnosis and differential diagnosis. In the future, we will include healthy populations and expand the sample size to explore VOCs biomarkers that distinguish patients from healthy populations for use in high-risk population surveys. 2) This study uses GC-IMS to detect VOCs in patients’ serum and analyze their characteristic peak intensity. The peak intensity can be directly read from the spectrum, which can quickly provide intuitive information on the relative content of compounds, but it may not be possible to accurate quantification. These may be the advantage and disadvantage of GC-IMS in detecting VOCs. GC-IMS had a high ability to separate complex components, and the ultra sensitivity of ion migration spectroscopy allows it to detect very small intensity volatile organic compounds. It had the advantages of fast analysis speed and no need for complex sample pretreatment. However, its database were relatively small. Compared with gas chromatography-mass spectrometry (GC-MS), the compound database of GC-IMS were not complete enough, and its ability to identify unknown compounds was relatively weak. Additionally, the accuracy of quantitative analysis needs to be improved (30); Although GC-MS had high resolution and accurate quantitative analysis, it can only be used for the separation and identification of low molecular weight (about 50 - 600 Da) and volatile compounds, and the analysis time was long. The sample pretreatment requirements were high, and chemical derivatization and other pretreatment were needed to detect polar, non thermal, and non-volatile metabolites. In addition, the instrument cost and maintenance cost were high (31). Selected ion flow tube mass spectrometry (SIFT-MS) can perform real-time and continuous monitoring of samples. Although the maintenance cost of this instrument was relatively low, it separates fewer volatile organic compounds (32). ENOSE detection was simple, fast, and inexpensive, but its sensitivity was limited and may be affected by environmental interference, making it impossible to separate individual volatile organic compound components (33). We will also conduct further high-precision quantitative analysis by selecting appropriate internal standards and analytical methods. 3) It was necessary to further explore the correlation mechanism between changes in endogenous VOCs and the occurrence and development of BTC or BBD.

## 5 Conclusion

We first used GC-IMS to analyze VOCs in the serum of BTC and BBD patients. Six machine learning methods, including RF, SVM, LDA, KNN, LASSO, and XGBoost, were used to construct and evaluate diagnostic prediction models for patients. Four VOCs were identified, and the model based on 4-VOC showed good sensitivity and specificity, which may be a new biomarker for distinguishing the diagnosis of BBD and BTC patients. In addition, we also constructed and evaluated the diagnostic performance of model in cholangiocarcinoma and benign biliary diseases, which provided new ideas for the differential diagnosis of biliary tract diseases

## Data Availability

The original contributions presented in the study are included in the article/**Supplementary Material**. Further inquiries can be directed to the corresponding authors.

## Supplementary material

Supplementary Figure 1 The fingerprint spectra of 14VOCs characteristic peak in patients.
