# Supplementary figures and images for: Identifying novel biomarkers for biliary tract cancer based on volatile organic compounds analysis and machine learning
Source: Front Oncol. 2025 Apr 24;15:1572460. doi: 10.3389/fonc.2025.1572460 (PMC12058901; doi:10.3389/fonc.2025.1572460)

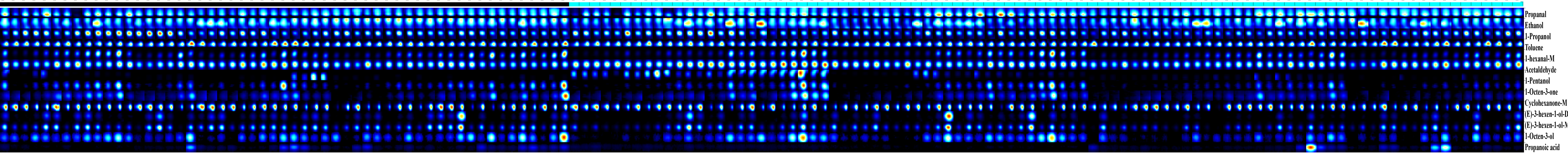

Supplement: Supplementary Figure 1 — The fingerprint spectra of 14VOCs characteristic peak in patients. [file Image1.jpeg]
