# Supplementary material for: Identifying novel biomarkers for biliary tract cancer based on volatile organic compounds analysis and machine learning
Source: Front Oncol. 2025 Apr 24;15:1572460. doi: 10.3389/fonc.2025.1572460 (PMC12058901; doi:10.3389/fonc.2025.1572460)
Supplement: Supplementary file 2 [file Table1.docx]

Supplementary Table S1. 40-VOC peaks were detected based on retention indices and drift times.

| **Compound** | **CAS#** | **Formula** | **MW** | **RI** | **Rt [sec]** | **Dt [a.u.]** | **Frequency(%)** |
| --- | --- | --- | --- | --- | --- | --- | --- |
| 2-ethyl hexanol-M | C104767 | C8H18O | 130.2 | 1458.5 | 405.473 | 1.42486 | 100 |
| 2-ethyl hexanol-D | C104767 | C8H18O | 130.2 | 1458.5 | 405.473 | 1.80038 | 47.9 |
| Propanoic acid | C79094 | C3H6O2 | 74.1 | 1516.7 | 500.472 | 1.11938 | 99.4 |
| Acetic acid | C64197 | C2H4O2 | 60.1 | 1436.4 | 374.371 | 1.06704 | 100 |
| 1-Octen-3-ol | C3391864 | C8H16O | 128.2 | 1421.2 | 354.43 | 1.16111 | 98.7 |
| ( E)-3-hexen-1-ol-M | C928972 | C6H12O | 100.2 | 1371.1 | 295.713 | 1.25794 | 100 |
| ( E)-3-hexen-1-ol-D | C928972 | C6H12O | 100.2 | 1371.1 | 295.713 | 1.54016 | 84.8 |
| 1-nonanal | C124196 | C9H18O | 142.2 | 1359.5 | 283.527 | 1.48759 | 100 |
| Hexyl propanoate | C2445763 | C9H18O2 | 158.2 | 1311.1 | 238.048 | 1.43545 | 66.5 |
| 1-Octen-3-one | C4312996 | C8H14O | 126.2 | 1285.6 | 218.921 | 1.27623 | 100 |
| Cyclohexanone-M | C108941 | C6H10O | 98.1 | 1267.7 | 209.077 | 1.15581 | 100 |
| Cyclohexanone-D | C108941 | C6H10O | 98.1 | 1266.1 | 208.233 | 1.45285 | 100 |
| 1-Pentanol | C71410 | C5H12O | 88.1 | 1232.6 | 191.075 | 1.2575 | 98.7 |
| Isobutyl 3-methylbutyrate | C589593 | C9H18O2 | 158.2 | 1183.7 | 168.523 | 1.3869 | 100 |
| Allyl sulfide-M | C592881 | C6H10S | 114.2 | 1121.2 | 143.086 | 1.11954 | 100 |
| Allyl sulfide-D | C592881 | C6H10S | 114.2 | 1120.5 | 142.827 | 1.32297 | 100 |
| 1- butanol | C71363 | C4H10O | 74.1 | 1134.1 | 148.018 | 1.18202 | 100 |
| Toluene | C108883 | C7H8 | 92.1 | 1058.1 | 124.327 | 1.01862 | 100 |
| 1-Propanol | C71238 | C3H8O | 60.1 | 1038.8 | 120.126 | 1.11427 | 100 |
| Pentanal-M | C110623 | C5H10O | 86.1 | 1025.2 | 117.261 | 1.19738 | 100 |
| Pentanal-D | C110623 | C5H10O | 86.1 | 1023.4 | 116.879 | 1.42318 | 100 |
| 2-Pentanone | C107879 | C5H10O | 86.1 | 992.2 | 110.577 | 1.36829 | 100 |
| Ethanol | C64175 | C2H6O | 46.1 | 954 | 104.085 | 1.11897 | 100 |
| 2-propanone | C67641 | C3H6O | 58.1 | 885.5 | 93.582 | 1.11584 | 100 |
| Propanal | C123386 | C3H6O | 58.1 | 839.3 | 87.089 | 1.03587 | 100 |
| Methanol | C67561 | CH4O | 32 | 923.8 | 99.311 | 0.97628 | 31.0 |
| Dimethyl sulfide | C75183 | C2H6S | 62.1 | 842.1 | 87.471 | 0.9559 | 96.8 |
| Acetaldehyde | C75070 | C2H4O | 44.1 | 789.4 | 80.596 | 0.97158 | 95.6 |
| 2-Propanol | C67630 | C3H8O | 60.1 | 932.4 | 100.647 | 1.24285 | 100 |
| Heptanal | C111717 | C7H14O | 114.2 | 1169.8 | 162.519 | 1.35105 | 100 |
| 3-Pentanol | C584021 | C5H12O | 88.1 | 1112.8 | 139.986 | 1.21776 | 100 |
| 1-Penten-3-ol | C616251 | C5H10O | 86.1 | 1144.8 | 152.207 | 0.93551 | 67.1 |
| 1-hexanal-M | C66251 | C6H12O | 100.2 | 1089.7 | 131.774 | 1.28832 | 100 |
| 1-hexanal-D | C66251 | C6H12O | 100.2 | 1090.3 | 131.965 | 1.5596 | 71.5 |
| Unidentified 1 | - | - | - | 817.7 | 84.225 | 1.3338 | 97.5 |
| Unidentified 2 | - | - | - | 854.6 | 89.19 | 1.55176 | 95.6 |
| Unidentified 3 | - | - | - | 865.6 | 90.717 | 1.68974 | 86.7 |
| Unidentified 4 | - | - | - | 940.9 | 101.984 | 1.68347 | 94.9 |
| Unidentified 5 | - | - | - | 934.8 | 101.029 | 1.86537 | 77.8 |
| Unidentified 6 | - | - | - | 1142.8 | 151.443 | 1.24128 | 100 |
